# Supplementary material for: Development of a High-Density 665 K SNP Array for Rainbow Trout Genome-Wide Genotyping
Source: Front Genet. 2022 Jul 18;13:941340. doi: 10.3389/fgene.2022.941340 (PMC9340366; doi:10.3389/fgene.2022.941340)
Supplement: Supplementary file 1 [file DataSheet2.PDF]

```
#!/usr/bin/env python3

import argparse
import vcf
import sys
import gzip

################################################################################
###################################################################
#
# COMMAND LINES USED TO FILTER THE DIFFERENT VCF FILE OF OUR STUDYs
#
# * Filtering the merged USDA dataset to keep :
#          - nuclear chromosome only
#      - bi-allelic variant
#      - MAF > 1% and keep MAF in INFO field using MAF_USDA tag
#
#       python3 script/vcf_filter.py --nuclear-only --bi-allelic --discthr 100
--hetthr 85 --missgtthr 100 --mafthr 1.0 --maf-info MAF_USDA \
#                                                                   -i
Results/USDA.vcf -o Results/USDA_MAF1_tagged.vcf;
#       bgzip Results/USDA_MAF1_tagged.vcf; bcftools view -f PASS -Oz -o
Results/USDA_MAF1.vcf.gz Results/USDA_MAF1_tagged.vcf.gz
#
#
# * Filtering the first INRAE dataset to keep only :
#          - on nuclear and mitochondrial chromosome only
#          - bi-allelic variants
#          - discordance rate (between line replicates) <=10%
#          - heterozygosity <= 1/19
#          - fully genotyped data --missgtthr 0
#          - MAF > 0% (--nonvar-filter)
# in this specific use case, only one replicate per isogenic line is returned.
This is hardly coded in the script.
#
#       python3 script/vcf_filter.py -i Results/Genomyks_SNP_simp.vcf -o
Results/Genomyks_SNP_simp_taggedd_noMAF.vcf --mafthr 0 --nuclear-only
--nonvar-filter --bi-allelic --discthr 10 --hetthr 1 --missgtthr 0 ;  bgzip
Results/Genomyks_SNP_simp_taggedd_noMAF.vcf
#
#
# * Filtering the merged INRAE dataset to keep :
#       - nuclear and mitochondrial chromosome only
#       - bi-allelic variant
#       - MAF > 1% and keep MAF in INFO field using MAF_INRA tag
#
#       python3 script/vcf_filter.py --nuclear-only --bi-allelic --discthr 100
--hetthr 79 --missgtthr 100 --mafthr 1.0 --maf-info MAF_INRA \
#                                                                   -i
Results/INRA.vcf -o Results/INRA_MAF1_tagged.vcf;
#       bgzip Results/INRA_MAF1_tagged.vcf; bcftools view -f PASS -Oz -o
Results/INRA_MAF1.vcf.gz Results/INRA_MAF1_tagged.vcf.gz
#
################################################################################
```

```
##################################################################


################################################################################
##################################################################
#
# FUNCTION
#
################################################################################
##################################################################

def is_gzip(file):
    """
    @return: [bool] True if the file is gziped.
    @param file : [str] Path to processed file.
    """
    is_gzip = None
    FH_input = gzip.open( file, 'rt' )
    try:
        FH_input.readline()
        is_gzip = True
    except:
        is_gzip = False
    finally:
        FH_input.close()
    return is_gzip

def process (params):

    FH_in=None
    if is_gzip(params.in_vcf):
        FH_in = vcf.Reader(gzip.open(params.in_vcf,'rt'))
    else:
        FH_in = vcf.Reader(open(params.in_vcf, 'rt'))

    ## add filter metadata
    # FILTER
    if params.nuclear_only:
        FH_in.filters["NW_CHR"]=vcf.parser._Filter("NW_CHR", "filtered out
variants on non nuclear chromosome")
    if params.nonvar_filter:
        FH_in.filters["unVar"]=vcf.parser._Filter("unVar", "filtered out non
variants site (all 1/1)")
    if params.discthr < 100:
        FH_in.filters["DISC"]=vcf.parser._Filter("DISC", "filtered out variants
with discordance genotype rate from replicate samples > "+str(params.discthr))
    if params.mafthr > 0:
        FH_in.filters["low_MAF"]=vcf.parser._Filter("low_MAF", "filtered out
variants called on dereplicated samples with MAF < "+str(params.mafthr))
    if params.bi_allelic:
        FH_in.filters["BI-AL"]=vcf.parser._Filter("BI-AL", "filtered out
variants called on dereplicated samples with more than 2 alleles")
    if params.missgtthr < 0:
        FH_in.filters["MISSGT"]=vcf.parser._Filter("MISSGT", "filtered out
```

```
variants called on dereplicated samples with more than "+str(params.missgtthr)+"
missing genotype")
    if params.hetthr > 0:
        FH_in.filters["high_HZ"]=vcf.parser._Filter("high_HZ", "filtered out
variants alled on dereplicated samples with more than "+str(params.hetthr)+"
heterozygote genotypes")
    # INFO
    FH_in.infos[params.maf_info] = vcf.parser._Info(params.maf_info,".",
"String", "MAF computed for the corresponding SNP on selected samples","","")

    FH_out = vcf.Writer(open(params.out_vcf, 'w'), FH_in)
    # recover samples replicates
    # {'R25_Genomyks': '', 'L28_Genomyks': '', 'X22_Genomyks': '',
'N33_Genomyks': '', 'A32_Genomyks': '', 'X34_Genomyks': '', 'G17': '',
'B03_Genomyks': '', 'X35_Genomyks': '',
    # 'AP2_Genomyks': 'AP2', '696': '695', 'B45_Genomyks': 'B45',
'A03_Genomyks': 'A03', 'B57_Genomyks': 'B57', 'AB1_Genomyks': 'AB1',
'A22_Genomyks': 'A22', 'R23_Genomyks': 'R23', 'A02_Genomyks': 'A02',
'A36_Genomyks': 'A36'}
    replicates = dict()
    if params.discthr < 100:
        replicates  = {'R25_Genomyks': '', 'L28_Genomyks': '', 'X22_Genomyks':
'', 'N33_Genomyks': '', 'A32_Genomyks': '', 'X34_Genomyks': '', 'G17': '',
'B03_Genomyks': '', 'X35_Genomyks': '',  'AP2_Genomyks': 'AP2', '696': '695',
'B45_Genomyks': 'B45', 'A03_Genomyks': 'A03', 'B57_Genomyks': 'B57',
'AB1_Genomyks': 'AB1', 'A22_Genomyks': 'A22', 'R23_Genomyks': 'R23',
'A02_Genomyks': 'A02', 'A36_Genomyks': 'A36'}
    for sample in FH_in.samples:
        if sample not in replicates.keys() and sample not in
replicates.values():
            replicates[sample] = ""

    # parsing record
    for record in FH_in:
        # compute discordance rate
        # record dereplicates samples alleles count
        # store dereplicates samples missing genotype
        # store dereplicates samples heterozygote genotype

        r = 0   # replicates genotyped
        d = 0   # replicates discordant
        alleles_count = dict()
        miss = 0
        hz = 0
        for sample in replicates:
            gt1 = record.genotype(sample).data.GT
            # replicates discordance rate
            if replicates[sample] != "":
                gt2 = record.genotype(replicates[sample]).data.GT
                if gt1 != "./." and gt2 != "./.":
                    r += 1
                    if gt1 != gt2:
                        d+=1
```

```
            # allele count
            a1 = gt1.split('/')[0]
            a2 = gt1.split('/')[1]
            if a1 not in alleles_count:
                alleles_count[a1]=0
            if a2 not in alleles_count:
                alleles_count[a2]=0
            alleles_count[a1]+=1
            alleles_count[a2]+=1

            # missing genotype
            if gt1 == "./.":
                miss +=1

            # hz genotype
            if a1 != a2:
                hz += 1

        record.FILTER = list()

        # filter out non nuclear chromosome
        if record.CHROM.startswith("NW") and params.nuclear_only:
            record.FILTER.append("NW_CHR")

        # filter on replicates discordance rate
        if r > 0 and d*100/r > params.discthr:
            record.FILTER.append("DISC")

        # compute and store MAF in INFO field
        # filter on MAF
        if "." in alleles_count:
            alleles_count.pop(".")

        min_all = 0
        tot_all = 0
        maf = 0.0
        if len(list(alleles_count.keys())) > 0:
            min_all = min(alleles_count.values())
            tot_all = sum(alleles_count.values())

        if tot_all > 0 :
            maf = round(min_all * 100.0 / tot_all, 3 )

        if maf == 100:
            maf = 0
        record.INFO[params.maf_info] = maf
        if maf == 0 and params.nonvar_filter:
            record.FILTER.append("unVar")
        elif maf < params.mafthr :
            record.FILTER.append("low_MAF")

        # correct alt allel if necessary (we do not take into account all
samples)
        # filter on bi-allelism
```

```
        #~ print(record)
        #~ print(alleles_count)
        if len(alleles_count) > 2 and params.bi_allelic:
            record.FILTER.append("BI-AL")
        if '0' in alleles_count:
            alleles_count.pop('0')

        # filter on missing
        if miss > params.missgtthr:
            record.FILTER.append("MISSGT")

        #filter on hz rate
        if hz > params.hetthr:
            record.FILTER.append("high_HZ")

        # write output file
        FH_out.write_record(record)

################################################################################
#####################################################################
#
# MAIN
#
################################################################################
#####################################################################
if __name__ == "__main__":
    parser = argparse.ArgumentParser( description='Completely filters a vcf for
SNPs and applies them \"PASS\" when they respect all given criteria.' )
    parser.add_argument( '--nuclear-only', default=False, action='store_true',
help="Keep only SNP on nuclear chromosome")
    parser.add_argument( '--nonvar-filter', default=False, action='store_true',
help="Keep only SNP with MAF>0, remove non varant site (all 1/1)")
    parser.add_argument( '--bi-allelic', default=False, action='store_true',
help="Keep only bi-allelic SNP")
    parser.add_argument( '--discthr', type=float, default=10.0, help="Percentage
of discordance rate for which the SNP has to be lower or equal than [Default:
%(default)s]")
    parser.add_argument( '--hetthr', type=int, default=1, help="over all
samples, number of maximum authorized samples to be heterozygous [Default:
%(default)s]")
    parser.add_argument( '--missgtthr', type=int, default=0, help="over all
samples, maximum number of authorized missing genotypes  [Default:
%(default)s]")
    parser.add_argument( '--mafthr', type=float, default=5.0, help="strict
minimum MAF threshold  [Default: %(default)s]")
    parser.add_argument( '--maf-info', type=str, default="MAF", help="MAF will
be stored in INFO fiel with this name  [Default: %(default)s]")
    # input
    group_input = parser.add_argument_group( 'Inputs' )
    group_input.add_argument( '-i', '--in-vcf', required=True, help="vcf file
containing variants to filter" )
    #     Outputs
    group_output = parser.add_argument_group( 'Outputs' )
    group_output.add_argument( '-o','--out-vcf', required=True, help="vcf output
```

```
file for SNPs with filters annotation" )
    args = parser.parse_args()

    process(args)
```
