# Supplementary material for: Development of a High-Density 665 K SNP Array for Rainbow Trout Genome-Wide Genotyping
Source: Front Genet. 2022 Jul 18;13:941340. doi: 10.3389/fgene.2022.941340 (PMC9340366; doi:10.3389/fgene.2022.941340)
Supplement: Supplementary file 2 [file DataSheet4.PDF]

```
#R script for final SNP selection for the HD trout chip
library(tidyverse)

#read the final set of SNPs to be proposed to Thermo Fisher in order to get only
665K SNPs (by removing SNPs in too dense regions)
listSNP <- read.table(file="final_snp_trimN.tsv",header=TRUE, sep="\t")

################################################################################
####################################################################

#       input file include the following columns:
#               Chromosome      NC_035077.1
#               Position        1009
#               ID      COM_NC_035077.1_1009
#               ID_Affx .
#               source  .
#               allele_consistency      .
#               50bp_before
AAACCCCACCTGGAGAAGCCCCTTCAGACCAAGCGTACCTCGATTACCAA
#               alleles [G/T]
#               50bp_after
AGGGCTAAGGTTTTAGTAGAGCCCATGCGTACCTTCAGTATAAGCTAAGG
#               MAF_INRA        25.0
#               MAF_USDA        19.565
#               35bp_before_score       65.8
#               35bp_before_coverage    100.0
#               35bp_before_identity    100.0
#               35bp_after_score        65.8
#               35bp_after_coverage     100.0
#               35bp_after_identity     100.0
#               nb_HZ   .
#
################################################################################
####################################################################
listSNP$Chromosome <- as.character(listSNP$Chromosome)
listSNP$ID <- as.character(listSNP$ID)
listSNP$ID_Affx <- as.character(listSNP$ID_Affx)

listchr <-  read.table(file="corres_omy_chr_ncbi.csv",header=TRUE, sep=";")
listchr$chr <- as.character(listchr$chr)
#content of file "corres_omy_chr_ncbi.csv"
#omy    chr
#1      NC_035077.1
#2      NC_035078.1
#3      NC_035079.1
#4      NC_035080.1
#5      NC_035081.1
#6      NC_035082.1
#7      NC_035083.1
#8      NC_035084.1
#9      NC_035085.1
#10     NC_035086.1
#11     NC_035087.1
#12     NC_035088.1
```

```
#13     NC_035089.1
#14     NC_035090.1
#15     NC_035091.1
#16     NC_035092.1
#17     NC_035093.1
#18     NC_035094.1
#19     NC_035095.1
#20     NC_035096.1
#21     NC_035097.1
#22     NC_035098.1
#23     NC_035099.1
#24     NC_035100.1
#25     NC_035101.1
#26     NC_035102.1
#27     NC_035103.1
#28     NC_035104.1
#29     NC_035105.1

data <- left_join(listSNP,listchr, by =c("Chromosome"="chr")) %>%
mutate(omy=replace_na(omy, 30), mbpos=Position/1000000)
data$omy <- as.factor(data$omy)

# Counting the number of SNPs per 100kb
data1 <- data %>% mutate(classe=cut(data$mbpos, breaks = seq(0,100,0.1))) %>%
mutate(classe_omy = paste0(omy, "_",classe))
data1$omy <- as.character(data1$omy)

data_counting <- as.data.frame(table(data1$classe_omy))
data_counting <- data_counting %>% arrange(desc(Freq))
data_counting <- data_counting %>% separate(Var1, c("omy","classe"), sep="_")
data_countinge$omy <- as.character(data_counting$omy)

data_freq <- inner_join(data1,data_counting, by =c("omy","classe")) %>%
arrange(desc(Freq)) %>% select(-c("allele_consistency","classe_omy"))
data_freq$MAF_INRA<- as.numeric(as.character(data_freq$MAF_INRA))
data_freq$MAF_USDA<- as.numeric(as.character(data_freq$MAF_USDA))

# Keeping SNPs if (there are present in the 57K chip with a sufficient MAF) OR
(if there is less than 31 SNPs per 100 kb) OR if either MAF is at least 15% in
one dataset (INRA or USDA) when there is more than 30 SNPs within 100kb
data2 <- data_freq %>%
            filter(ID_Affx !="." & (MAF_INRA > 4.999 | MAF_USDA > 4.999 ) | Freq
< 31 | (Freq > 30 & (MAF_INRA > 14.999 | MAF_USDA > 14.999 ))) %>%
            select(-c(source, Freq, classe))
data2  <- arrange(data2,omy)
write.table(data2,"List_665K.csv", sep = ";", col.names = TRUE, row.names =
FALSE,quote = FALSE)
```
