# Supplementary material for: Development of a High-Density 665 K SNP Array for Rainbow Trout Genome-Wide Genotyping
Source: Front Genet. 2022 Jul 18;13:941340. doi: 10.3389/fgene.2022.941340 (PMC9340366; doi:10.3389/fgene.2022.941340)
Supplement: Supplementary file 3 [file DataSheet3.PDF]

```
#!/usr/bn/python

import argparse

#####################################################################################
###################################################################
#
# COMMAND LINE USED TO TRIM SNP FLANKING SEQUENCES THAT INCLUDE STRECH OF N AND
EXCLUDE THEM IF REMAINING LENGTH ARE BELLOW 20.
#
#       python2.7 script/trimN.py -i Results/snp.tsv -o Results/snp_trimN.tsv -m
Results/marker_excluded.tsv -v temp/variant_excluded.tsv
#
#       input file include the following columns:
#               Chromosome      NC_035077.1
#               Position        1009
#               ID      COM_NC_035077.1_1009
#               ID_Affx .
#               source  .
#               allele_consistency      .
#               50bp_before
AAACCCCACCTGGAGAAGCCCCTTCAGACCAAGCGTACCTCGATTACCAA
#               alleles [G/T]
#               50bp_after
AGGGCTAAGGTTTTAGTAGAGCCCATGCGTACCTTCAGTATAAGCTAAGG
#               MAF_INRA        25.0
#               MAF_USDA        19.565
#               35bp_before_score       65.8
#               35bp_before_coverage    100.0
#               35bp_before_identity    100.0
#               35bp_after_score        65.8
#               35bp_after_coverage     100.0
#               35bp_after_identity     100.0
#               nb_HZ   .
#
#####################################################################################
###################################################################

def trim(seq,end):
    """
    @Summary : trim sequence to keep the longuest extremities until the stretch
of N
    @param seq : [str] string to trim
    @param end : [str] extremities to keep : begin or end
    @return the trimmed seq
    """

    # sequence before SNP, keep the closest part of the SNP so the end
    if end == "end" and "NNNN" in seq:
        return seq[seq.rfind("NNNN")+4 :]
    elif end == "begin" and "NNNN" in seq:
        return seq[ : seq.find("NNNN")]
    else:
        return seq
```

```
def process(params):

    FH_in = open(params.in_tsv)
    FH_out = open(params.out_tsv ,"w")
    FH_markExcl = open(params.mark_excl_tsv ,"a")
    FH_varExcl = open(params.var_excl_tsv ,"a")

    for line in FH_in:
        if line.startswith("#Chromosome"):
            FH_out.write(line)
        else :
            line_list = line.split()
            before = line_list[6]
            after = line_list[8]
            if "NNNN" in before:
                before = trim(before, "end")
            if "NNNN" in after:
                after = trim(after, "begin")
            line_list[6] = before
            line_list[8] = after
            if len(before) >= 20 and len(after) >= 20:
                FH_out.write("\t".join(line_list) + "\n")
            elif line_list[3] != ".":
                FH_markExcl.write("\t".join(line_list) + "\n")
            else:
                FH_varExcl.write("\t".join(line_list) + "\n")

    FH_in.close()
    FH_out.close()

################################################################################
##################################################################
#
# MAIN
#
################################################################################
##################################################################
if __name__ == "__main__":
    parser = argparse.ArgumentParser( description='trim to keep flanking
sequence with a maximum of 3 Ns' )
    # Input
    group_input = parser.add_argument_group( 'Inputs' )
    group_input.add_argument( '-i', '--in-tsv', required=True, help="TSV input
file with flanking sequence in column 7 and 9" )
    # Outputs
    group_output = parser.add_argument_group( 'Outputs' )
    group_output.add_argument( '-o','--out-tsv', required=True, help="TSV output
file with trim flanking sequences with a minimum of 20 bp" )
    group_output.add_argument( '-m','--mark_excl-tsv', required=True, help="TSV
output file excluded marker because trim flanking sequences are < 20" )
    group_output.add_argument( '-v','--var-excl-tsv', required=True, help="TSV
output file excluded new variant because trim flanking sequences are < 20" )
    args = parser.parse_args()
```

```
process(args)
```
