# Supplementary material for: Development of a High-Density 665 K SNP Array for Rainbow Trout Genome-Wide Genotyping
Source: Front Genet. 2022 Jul 18;13:941340. doi: 10.3389/fgene.2022.941340 (PMC9340366; doi:10.3389/fgene.2022.941340)
Supplement: Supplementary file 4 [file DataSheet1.PDF]

Supplementary Material

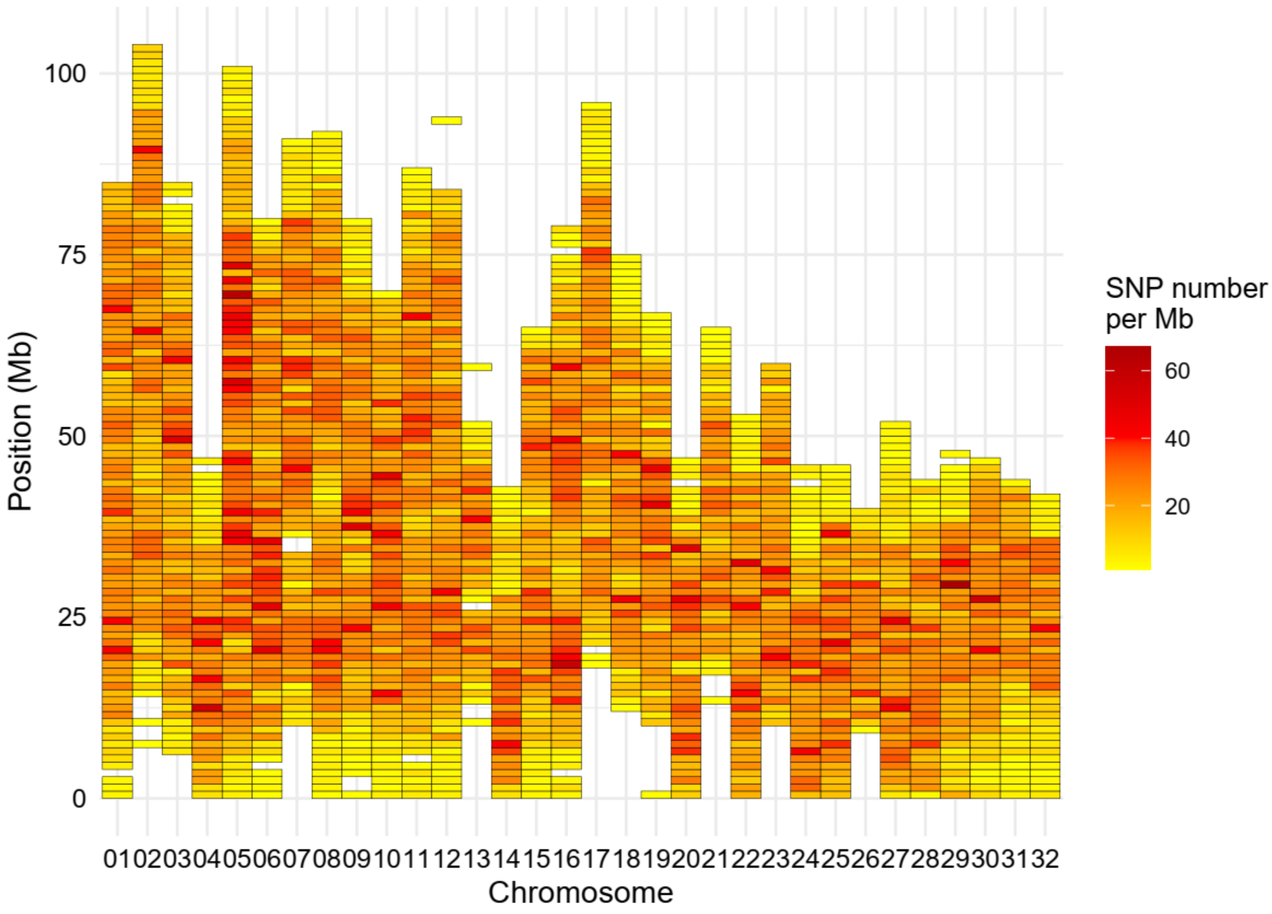

Supplementary Figure 1. Marker density per Mb for the LD Trout Affymetrix array with 38,948 SNPs positioned on the 32 chromosomes of the Arlee genome reference
